# Telomere length and its correlation with gene mutations in chronic lymphocytic leukemia in a Korean population

**DOI:** 10.1371/journal.pone.0220177

**Published:** 2019-07-23

**Authors:** Da Young Song, Jung-Ah Kim, Dajeong Jeong, Jiwon Yun, Sung-Min Kim, Kyumin Lim, Si Nae Park, Kyongok Im, Sungbin Choi, Sung-Soo Yoon, Dong Soon Lee

**Affiliations:** 1 Department of Laboratory Medicine, Seoul National University College of Medicine, Seoul, Korea; 2 Department of Laboratory Medicine, Chung-Ang University Hospital, Seoul, Korea; 3 Cancer Research Institute, Seoul National University College of Medicine, Seoul, Korea; 4 University of British Columbia, Vancouver, Canada; 5 Department of Internal Medicine, Seoul National University College of Medicine, Seoul, Korea; University of Newcastle, UNITED KINGDOM

## Abstract

Telomere length (TL) is a prognostic indicator in Caucasian chronic lymphocytic leukemia (CLL), but its significance in Asian CLL remains unknown. To investigate the prognostic significance of TL and its correlation with cytogenetic aberrations and somatic mutations, we analyzed TL measurements at the cellular level by interphase fluorescence *in situ* hybridization in patients with CLL in Korea. The present study enrolled 110 patients (41 females and 69 males) diagnosed with CLL according to the World Health Organization criteria (2001–2017). TLs of bone marrow nucleated cells at the single-cell level were measured by quantitative fluorescence *in situ* hybridization (Q-FISH) in 71 patients. The correlations of TL with clinical characteristics, cytogenetic aberrations, genetic mutations, and overall survival were assessed. The median value of mean TL in CLL patients (T/C ratio 7.46 (range 1.19–18.14) was significantly shorter than that in the normal controls (T/C ratio 15.28 (range 8.59–24.93) (*p* < 0.001). Shorter TLs were associated with complex karyotypes (*p* = 0.030), del(11q22) (*p* = 0.023), presence of deletion and/or mutation in *ATM* and/or *TP53* (*p* = 0.019), and *SH2B3* mutation (*p* = 0.015). A shorter TL was correlated with lower hemoglobin levels and adverse survival (mean TL < 9.35, *p* = 0.021). When the proportion of cells with extremely short TLs (< 7.61) was greater than 90%, CLL patients showed poor survival (*p* = 0.002). Complex karyotypes, *TP53* mutation, and the number of mutated genes were determined to be significant adverse variables by multivariable Cox analysis (*p* = 0.011, *p* = 0.002, and *p* = 0.002, respectively). TL was attrited in CLL, and attrited telomeres were correlated with adverse survival and other well-known adverse prognostic factors. We infer that TL is an independent adverse prognostic predictor in Korean CLL.

## Introduction

Telomeres are end complexes that protect eukaryotic chromosomes, and they are known to be related to aging-related disease and an increased or decreased risk of cancer [1]. Their erosion and fusion have a significant effect on many hematologic malignancies, including chronic lymphocytic leukemia (CLL) [2]. Telomere length (TL) has been reported to be a prognostic indicator in CLL [3–5]. Additionally, telomere dysfunction and short telomeres are related to patient survival, treatment requirements, and transformation to Richter syndrome in CLL [6, 7].

CLL is a rare disease in Asian countries, including Korea [8]. CLL shows relatively aggressive clinical behavior in the Korean population [9]. The characteristics of CLL, including cytogenetic and molecular characteristics and CD38 and ZAP-70 expression, are informative for prognostic and/or therapy determination [10]. Only a few reports on genomic changes in Asian CLL patients exist. We previously reported ethnic differences between Caucasian and Korean CLL. Mutation profiles and prognostic genes were different between Korean and Caucasian CLL, while cytogenetic aberrations were found to be similar between the two groups [11]. Therefore, we questioned whether a shortened TL is associated with an adverse prognosis in Korean CLL.

The aim of this study was to clarify the relationship between TL and genomic mutations and to determine the effect of TL on the prognosis of CLL in a Korean population. In addition, this study will compare the results to those of a Caucasian study to determine whether any differences in TL exist.

## Materials and methods

### Study populations

The study was reviewed and approved by the Institutional Review Board of Seoul National University Hospital (IRB No. 1307-090-505). The present study included 110 patients (41 females and 69 males) diagnosed with CLL. A series of 97 patients who had undergone bone marrow (BM) examination and were diagnosed with chronic lymphocytic leukemia/small lymphocytic lymphoma (CLL/SLL) between September 2001 and July 2017 at Seoul National University Hospital (SNUH) were selected for this study. Five patients who had been diagnosed with CLL/SLL prior to visiting SNUH were included in this group (n = 5). In another group of 13 patients who had undergone lymph node (LN) biopsies and had been diagnosed with CLL/SLL between April 1999 and October 2013 at Asan Medical Center (AMC), three patients had been diagnosed with CLL/SLL prior to visiting AMC. All of the patients in the study were Korean. Normal BM samples were collected from BM donors for staging of malignant lymphoma who were proven to have no evidence of malignant lymphoma by immunohistochemical study, immunoglobulin rearrangement study and TCR rearrangement study. Institutional Review Board (IRB) approval for residual samples was acquired (IRB No. 1307-090-505), and consent was obtained from each patient. The diagnosis of CLL/SLL was based on the World Health Organization (WHO, 2008) classification criteria and the 2008 International Workshop on Chronic Lymphocytic Leukemia-National Cancer Institute criteria (IWCLL-NCI). Fluorescence *in situ* hybridization (FISH) for IgH/CCND1 translocations was performed to confirm that the disease was not a leukemic phase of mantle cell lymphoma. Clinical staging was performed using the Binet staging system (classes A, B and C). This staging system is based on the hemoglobin (Hb) level and platelet (PLT) count as well as the number of involved enlarged lymphoid tissue areas (i.e., head and neck, axillae, and groin) and organomegaly.

Laboratory data, including age, sex, diagnosis and therapy start dates, complete blood count (CBC), BM morphology, BM CLL cell count percentage sorted by flow cytometry, cytogenetic analysis, and FISH, were obtained for each patient. Mononuclear cells from the initial BM aspirates of patients who underwent BM aspiration and biopsy at the time of diagnosis were fixed in Carnoy’s solution and stored at −70°C for FISH analysis.

### Bone marrow examination and leukemia-lymphoma marker study

Hematopathologists reviewed the Wright-stained BM smears and hematoxylin and eosin (H&E)-stained sections of BM trephine biopsies to determine the percentages and patterns of BM infiltration by lymphocytes. The median lymphoid cell percentage was 66% on aspiration (range, 5–98%). The median cellularity was 60% (range, 15–100%). ZAP-70 (Cell Marque, Rocklin, CA, USA) staining was performed on BM sections from 62 patients. The leukemia-lymphoma marker study was performed. The antibodies used were specific for TdT, CD2, CD3, CD5, CD7, CD10, CD19, CD20, CD22, CD23, FMC7, CD45, CytoCD3, CD56, Kappa, and Lambda (BD Biosciences, San Jose, CA, USA) and were measured using the Navios Cytometer (Beckman Coulter, Villepinte, France).

### Lymph node biopsy examination

A pathologist reviewed the formalin-fixed and paraffin-embedded LN sections. Immunohistochemistry staining was performed using antibodies specific for CD3, CD5, CD10, CD20, CD23, cyclin D1, BCL-6, and Ki-67 (all from Dako, Glostrup, Denmark).

### G-banding and fluorescence *in situ* hybridization

Cytogenetic studies using the standard G-banding technique on heparinized BM samples were performed as part of the diagnostic work-up. To stimulate B cells, tetradecanoylphorbol acetate (TPA; phorbol-12-myristate-13-acetate) was added, and the cells were cultured for 4 days. At least 20 cells in metaphase were analyzed whenever possible. Clonal abnormalities were defined as two or more cells with the same chromosomal gain or structural rearrangement or at least three cells with the same chromosome loss.

Common chromosomal abnormalities were investigated using commercial FISH probes. We used the following probes for enumeration of chromosome 12 and for detection of the 13q14.3, 17p13, and 11q22 deletions and the IgH/CCND1 translocation (to exclude mantle cell lymphoma): the LSI D13S319/LSI13q34/CEP12 Multi-Color Probe, LSI TP53 (17p13.1) SpectrumOrange Probe, Vysis IGH/CCND1 XT DF FISH Probe (all from Abbott Molecular/Vysis, Des Plaines, IL, USA), and XL ATM/TP53 Probe (Metasystems, GmbH, Altlussheim, Germany). Interphase FISH was performed on stored patient BM aspirate specimens. Slides were stained with FISH probes and counterstained with 4’, 6-diamidino-2-phenylindole (DAPI), and fluorescence signals were analyzed by fluorescence microscopy (Zeiss, Göttingen, Germany). The cut-off values for the deletion, amplification, or translocation of chromosomal regions were calculated based on the mean values of the normal controls and +3 standard deviations (bone marrow from 20 normal individuals). The cut-off values for patients were 1.50% for trisomy 12, 4.58% for 13q14.3 deletion, 7.39% for 17p13 deletion, and 5.59% for 11q22 deletion.

### Quantitative measurement of TL using interphase fluorescence *in situ* hybridization (Q-FISH)

As described in detail previously [12], quantitative FISH (Q-FISH) was performed using the Cy3-labeled Telomere PNA (peptide nucleic acid) FISH kit (DakoCytomation Denmark A/S, Glostrup, Denmark) and an FITC-labeled PNA probe for the centromere of chromosome 2 (kindly provided by Dako). One microliter of the chromosome 2 centromere probe was added to 9 μL of the telomere probe. The telomere and centromere Q-FISH hybridizations were performed according to the manufacturer’s instructions. Interphase Q-FISH images were captured using a Zeiss Axioplan 2 imaging microscope (Zeiss, Germany) equipped with ISIS software (MetaSystems). To measure TL, the ISIS-Telomere module (MetaSystems) was used as previously described. The software calculates a telomere/centromere (T/C) fluorescence intensity ratio, which is a measure of the TL, for each individual chromosome arm within each metaphase and interphase nucleus, as previously described. The T/C ratio was multiplied by 100 and used as the TL. At least 100 interphase nuclei were scanned for each patient. Considering the quantitative differences in mutational profiles and telomere lengths between peripheral blood and bone marrow [13–15], we performed TL analysis on normal bone marrow cells, as normal bone marrow cells are appropriate for comparison. We used normal BM samples from 23 healthy individuals for comparison.

### DNA extraction and targeted sequencing

DNA extraction was successful in 70 of the samples (28 samples of frozen BM mononuclear cells, 19 BM aspirate smears, and 23 formalin-fixed paraffin-embedded BM and LN biopsies). Sample quality was evaluated using the Agilent 2200 TapeStation System (Santa Clara, CA, USA). Only 48 of the 70 samples met the DNA quality control criteria for multigene targeted sequencing. DNA was extracted from frozen BM mononuclear cells using a MagNA Pure LC DNA Isolation Kit (Roche Diagnostics GmbH, Mannheim, Germany) with the MagNA Pure LC 2.0 System (Roche) according to the manufacturer’s instructions. DNA was extracted from the BM aspirate smear unstained slides using a QIAamp DNA Blood Mini Kit (Qiagen, Hilden, Germany) and Tissue Lysis Buffer (Qiagen). DNA was extracted from the FFPE BM biopsy samples using a WaxFree^™^ Kit (TrimGen Genetic Diagnostics; Sparks, MD, USA) according to the manufacturer’s instructions. All samples were stored at −20°C.

To gain insight into the genetic lesions that drive CLL, we manually prioritized 87 hematology malignancy-related genes and performed targeted sequencing. Of the 48 samples, two samples were subjected to whole-genome amplification. gDNA shearing to generate the standard library and the hybridization step targeting only exonic regions were performed by Celemics Inc. (Seoul, Korea). The final quality was assessed using the Agilent 2200 TapeStation System (Santa Clara, CA, USA). We sequenced the total target length of 259-kb regions using the paired-end 150-bp rapid-run sequencing mode on the Illumina HiSeq 2500 platform. We achieved over 10x coverage for greater than 97% of the targeted regions for each sample. The mean sequencing depth for the targeted regions (259 kb) was 231-fold (n = 48). Because a matched control sample was not included in this study, we applied a stringent variant selection pipeline to prioritize the high-confidence set of somatic mutations.

### Statistical analysis

Nonparametric analysis of covariance adjusting for age was performed to compare the TL between CLL and normal control groups. Correlation coefficients for the CBC profile, BM lymphocytes, Richter syndrome, ZAP70, cytogenetics and gene mutations, TL, and the percentage of cells with the shortest TL (STL%) were calculated by Kendall’s tau-b (Tb) correlation test. TLs between subgroups were compared using the Mann-Whitney test and Kruskal-Wallis rank sum test. Survival was estimated using the Kaplan-Meier (K-M) method, and differences between the survival curves and hazard ratios were analyzed using the Cox proportional hazards model and Harrell’s C-index. Factors with *p* values less than 0.2 in the univariate analysis were entered into the multivariate analysis. Overall survival (OS) was calculated from the date of diagnosis until the date of the last follow-up or death. Time-to-First-Treatment (TTT) analysis was calculated from the interval of time between the diagnosis and the date of first CLL treatment [16]. Statistical analyses were performed using R software (http://www.r-project.org), IBM SPSS Statistics Version 23.0. (Armonk, IBM Corp.), SAS 9.2 Version (SAS Institute Inc., Cary, NC, USA.), and STATA 15.0 (StataCorp. 2017. Stata Statistical Software: Release 15. College Station, TX: StataCorp LLC). A *p* value < 0.05 was considered statistically significant.

## Results

### Patient demographic information

The clinical features of the patients with CLL (n = 110) are summarized in Table 1. Of 110 patients, 69 (62.7%) were male, and 41 (37.3%) were female. The mean age was 61.6 years. The Binet stage distribution was as follows: stage A in 55.5% (61/110), stage B in 23.6% (26/110), and stage C in 20.9% (23/110) of the patients. The mean values of CBC profiles at the time of BM sample collection were 12.3 (± 2.2) (g/dL) Hb, 29,700 (± 42,300) (x/μL) white blood cells (WBCs), 22,600 (± 33,900) (x /μL) absolute lymphocyte count, and 172.3 (± 66.9) (x/μL) PLTs. The mean percentage of BM lymphocytes was 59.8% (± 26.4). During the study period, six patients (5.5%) transitioned to Richter syndrome; 4 patients progressed to diffuse large B cell lymphoma, 1 patient progressed to prolymphocytic leukemia, and 1 patient progressed to composite lymphoma of peripheral T cell lymphoma (unspecified) and large B cell lymphoma.

**Table 1:** Characteristics of the CLL Patients (n = 110)^a^.

Characteristics	n (%) or n / total n (%) or mean ± SD
Sex	
Male	69 (62.7%)
Female	41 (37.3%)
Age	61.6 ± 11.5
History of treatment	
Yes	29 (26.4%)
No	81 (73.6%)
Binet stage	
A	61 (55.5%)
B	26 (23.6%)
C	23 (20.9%)
Complete blood count at bone marrow sample collection	
WBCs (10^3^/μL)	29.7 ± 42.3
Absolute lymphocyte count (10^3^/μL)	22.6 ± 33.9
Hb (g/dL)	12.3 ± 2.2
Platelets (10^3^/μL)	172.3 ± 66.9
Bone marrow lymphocyte (%)	59.8 ± 26.4
Cytogenetic results	
Karyotype	
Normal karyotype	66/97 (68.0%)
Aberrant karyotype	31/97 (32.0%)
Complex karyotype (>3 abnormalities)	17/97 (17.5%)
Fluorescence *in situ* hybridization	
Trisomy 12	22/85 (25.9%)
del(13q)	34/83 (41.0%)
del(17p)	6/86 (7.0%)
del(11q22)	8/80 (10.0%)
*ATM* mutation	10/48 (20.8%)
*TP53* mutation	7/48 (14.6%)
*ATM* defect (del(11q22) and/or *ATM* mutation)	13/39 (33.0%)
*TP53* defect (del(17p13) and/or *TP53* mutation)	9/40 (22.5%)
Richter syndrome	6/110 (5.5%)

^a^ Depending on the scale of the characteristic and its distribution, either the absolute number n (percentage) or the mean (standard deviation SD) is given. If the available number of patients is less than the total number of patients (n = 110), then the absolute number / available number (percentage) is given.

Abbreviations: n, number; SD, standard deviation.

The OS associated with clinical characteristics, CBC, BM lymphocyte counts, cytogenetic abnormalities, and genetic mutations was analyzed. Significant differences in survival depending on age group (age > 63 vs. ≤ 63, *p* = 0.037), Hb (≤ 12.1/dL vs. > 12.1, *p* = 0.008), WBC count (≤ 6,700/μL vs. > 6,700/μL, *p* = 0.048), PLT count (≤ 94,000/μL vs. > 94,000/μL, *p* < 0.001), and BM lymphocytes (> 80.8% vs. ≤ 80.8%, *p* < 0.001) were found according to K-M survival analysis. Binet stage C was associated with lower OS than stages A and B (*p* = 0.018). Patients who progressed to Richter syndrome showed lower survival than the others (*p* < 0.001). On the other hand, in case of TTT analysis, significant differences in prognosis depending on age group (age > 60 vs. ≤ 60, *p* = 0.0), Hb (≤ 11.6/dL vs. > 11.6, *p* < 0.001), WBC count (≤ 10,140/μL vs. > 10,140/μL, *p* = 0.005), PLT count (≤ 104,000/μL vs. > 104,000/μL, *p* < 0.001), and PB lymphocytes (> 5,840/μL vs. ≤ 5,840/μL, *p* = 0.034) were found according to K-M survival analysis. Binet stage A was associated with higher OS than were stages B and C (*p* < 0.001). Other CBC profiles were not related to OS and TTT.

### Cytogenetic and genetic changes in CLL

In addition to a previous study, patients with available specimens were subjected to cytogenetic studies [11]. A cytogenetic study was performed using G-banding (97/110) and FISH (from 80 to 86 patients according to FISH type). Thirty-two percent (31/97) of the patients had an aberrant karyotype, and 17.5% (17/97) had a complex karyotype representing three or more chromosomal abnormalities. The FISH abnormalities were trisomy 12 in 25.9% (22/85) of patients, 13q14.3 deletion in 41.0% (34/83) of patients, 17p13 deletion in 7.0% (6/86) of patients, and 11q22 deletion in 10.0% (8/80) of patients. Among the patients with abnormal FISH results, 39 patients had 1 abnormality, 14 patients had 2 abnormalities, and 1 patient had 3 abnormalities. Genetic variations from a previous report were used for analysis and are described in the supplemental data.

### Distributions of TL in the CLL group and normal control group

**Fig 1:**
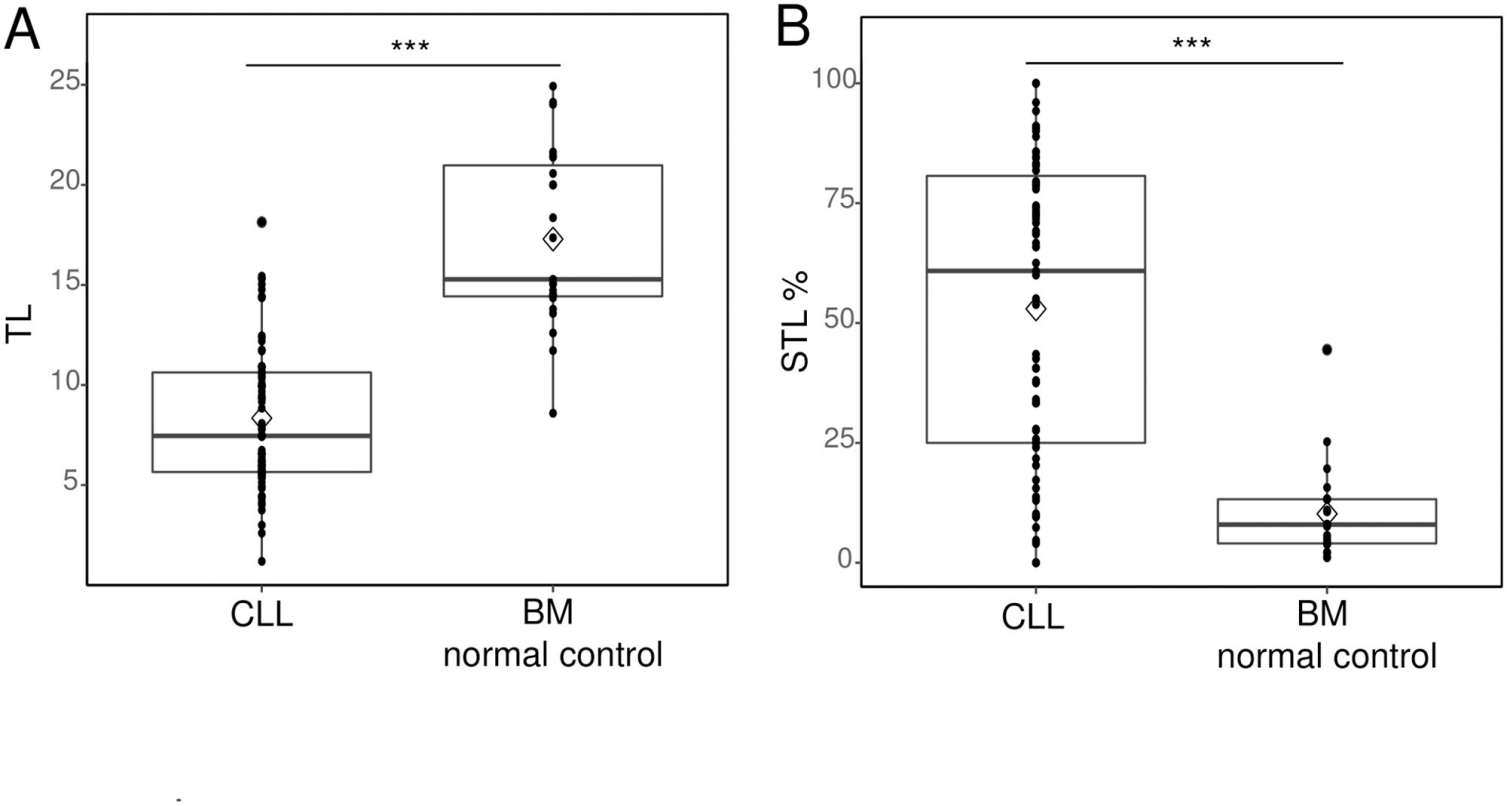
The TL and STL% of CLL patients. (A) The mean telomere lengths (TLs) were compared between CLL patients and bone marrow (BM) from normal controls. The CLL patients’ TLs were shorter than those in the normal BM samples with age adjustment (7.46 (range 1.19–18.14) in CLL patients; 15.28 (range 8.59–24.93) in normal BM control samples, median value). (B) The STL% was compared between the CLL patient group and normal BM control group. CLL patients had a higher STL% than the control group (52.95 (± 31.33) in the CLL group and 10.20 (± 9.65) in the normal control group). *** indicates *p* < 0.001 for comparison.

The TLs of BM nucleated cells from CLL patients (n = 71) and normal BM (n = 23) were compared (Fig 1A). The normal control group included healthy persons over 45 years of age, and the mean age was 60.9 years. TL was expressed as the T/C ratio. The average T/C ratio of analyzed cells per patient was expressed as the mean TL of the patient. The median value of mean TL was 7.46 (range 1.19–18.14) in CLL patients and 15.28 (range 8.59–24.93) in normal controls. With age adjustment, the TLs of CLL patients were significantly shorter than those of normal controls (p < 0.001). The TL of CLL patients was significantly shorter than that of normal controls (*p* < 0.001) after age adjustment. The TL of CLL patients was distributed shorter than normal controls regardless of age (S1 Fig).

**Fig 2:**
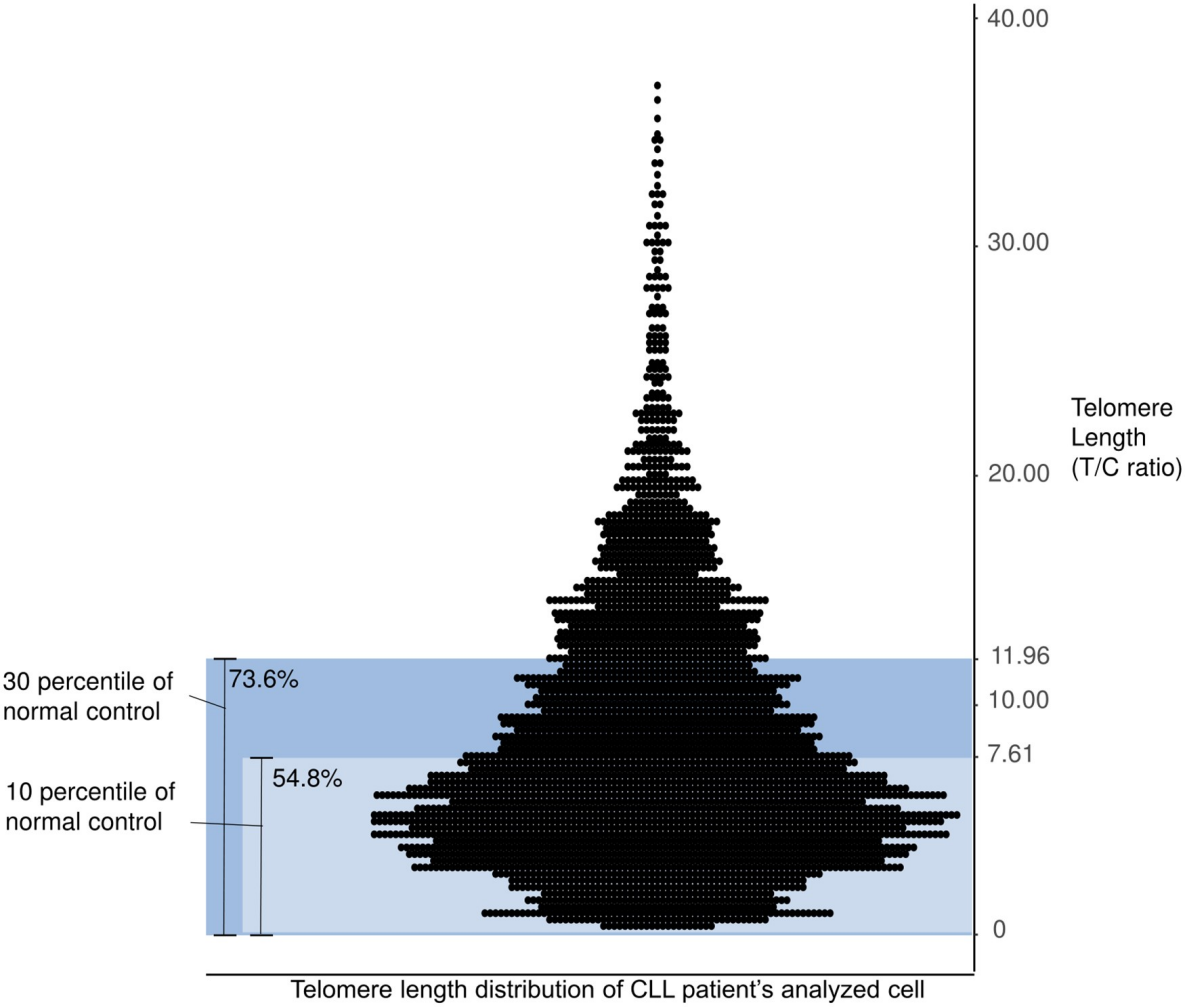
The TL distribution of CLL patients’ analyzed cells. The TLs of analyzed cells from CLL patients (n = 71) and the normal control percentiles are presented in the dot plot and bar. The TLs of CLL cells were generally biased to the lower percentile of the normal control group. Cells with TLs shorter than 7.61, the length of the 10^th^ percentile of a normal TL, accounted for 54.8% of CLL cells, and cells with a TL shorter than 11.96, the length of the 30^th^ percentile of a normal TL, accounted for 73.6% of CLL cells. CLL patients’ cells with a TL longer than 40.00 (T/C ratio) are not shown in this graph (n = 4, range 44.59–56.70).

To observe the proportion of cells with extremely short TLs in CLL, we analyzed the distribution profile of TL by pooling analyzed individual cells of CLL patients and normal control groups. We defined the STL as a TL shorter than 7.61 (T/C ratio), which is the lower 10^th^ percentile T/C value of cells in the normal control group. Therefore, the percentages of cells with the shortest telomeres (STL%) were calculated (the number of cells with STL/total number of analyzed cells in each CLL patient). The mean STL% was 53.0% (± 31.33) (range 0–100) in CLL, indicating that approximately half of the cells in CLL patients were distributed below the 10^th^ percentile of the normal control group (Fig 1B). The TLs of CLL cells were generally found in a lower percentile than the TLs in the normal control group and showed a skewed distribution. Cells with a TL shorter than 7.61, the length of the 10^th^ percentile of a normal TL, accounted for 54.8% of CLL cells, and cells with a TL shorter than 11.96, the length of the 30^th^ percentile of a normal TL, accounted for 73.6% of CLL cells (Fig 2).

### TL and its correlation with clinical characteristics

**Fig 3:**
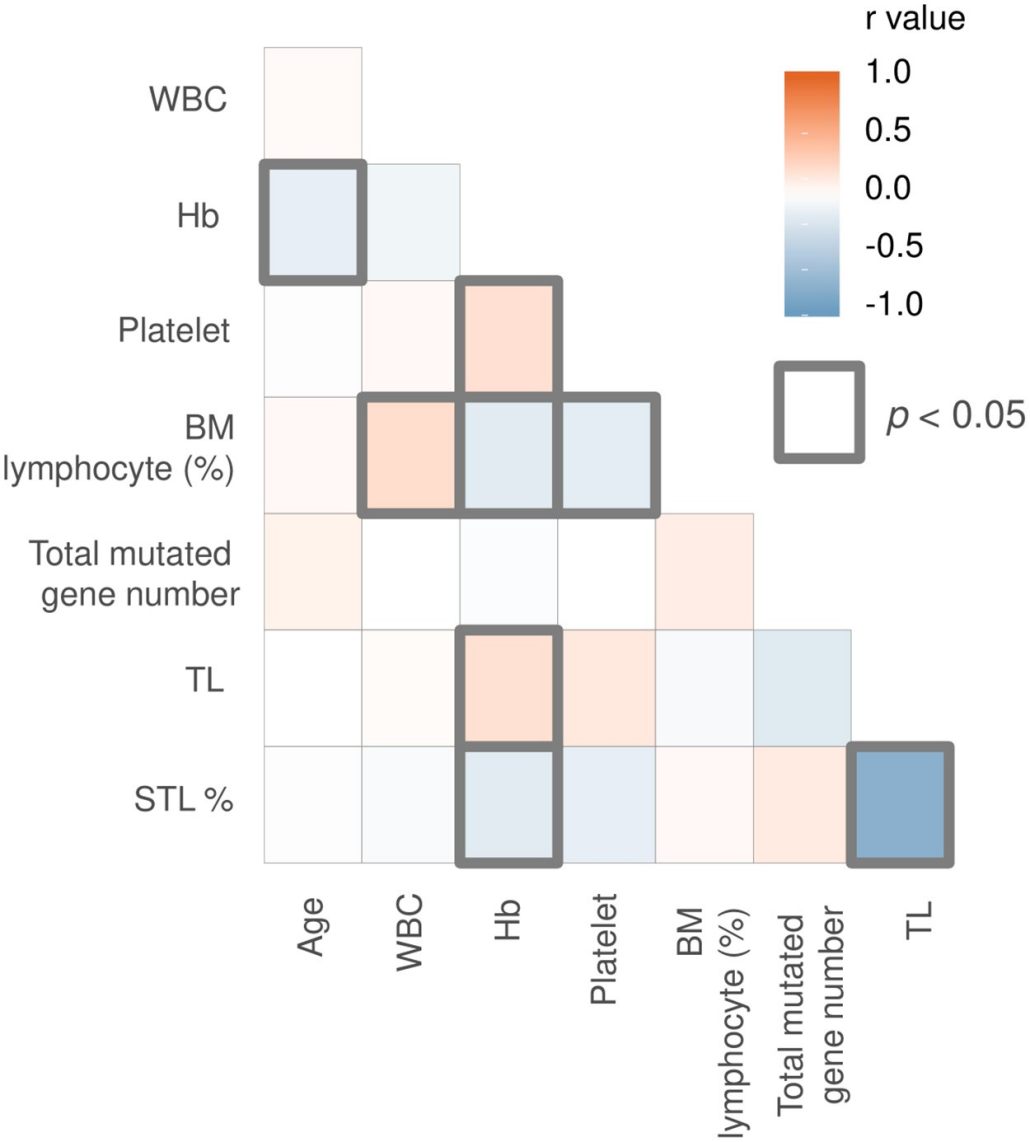
Correlation analysis of TL, the STL%, and clinical parameters. The graph shows that Hb had statistically significant correlations with TL (Tb = 0.188, *p* = 0.021) and the STL% (Tb = -0.207, *p* = 0.013). Significant correlations are marked as bold boxes. * Abbreviations: WBC, white blood cell; Hb, hemoglobin; BM, bone marrow.

**Fig 4:**
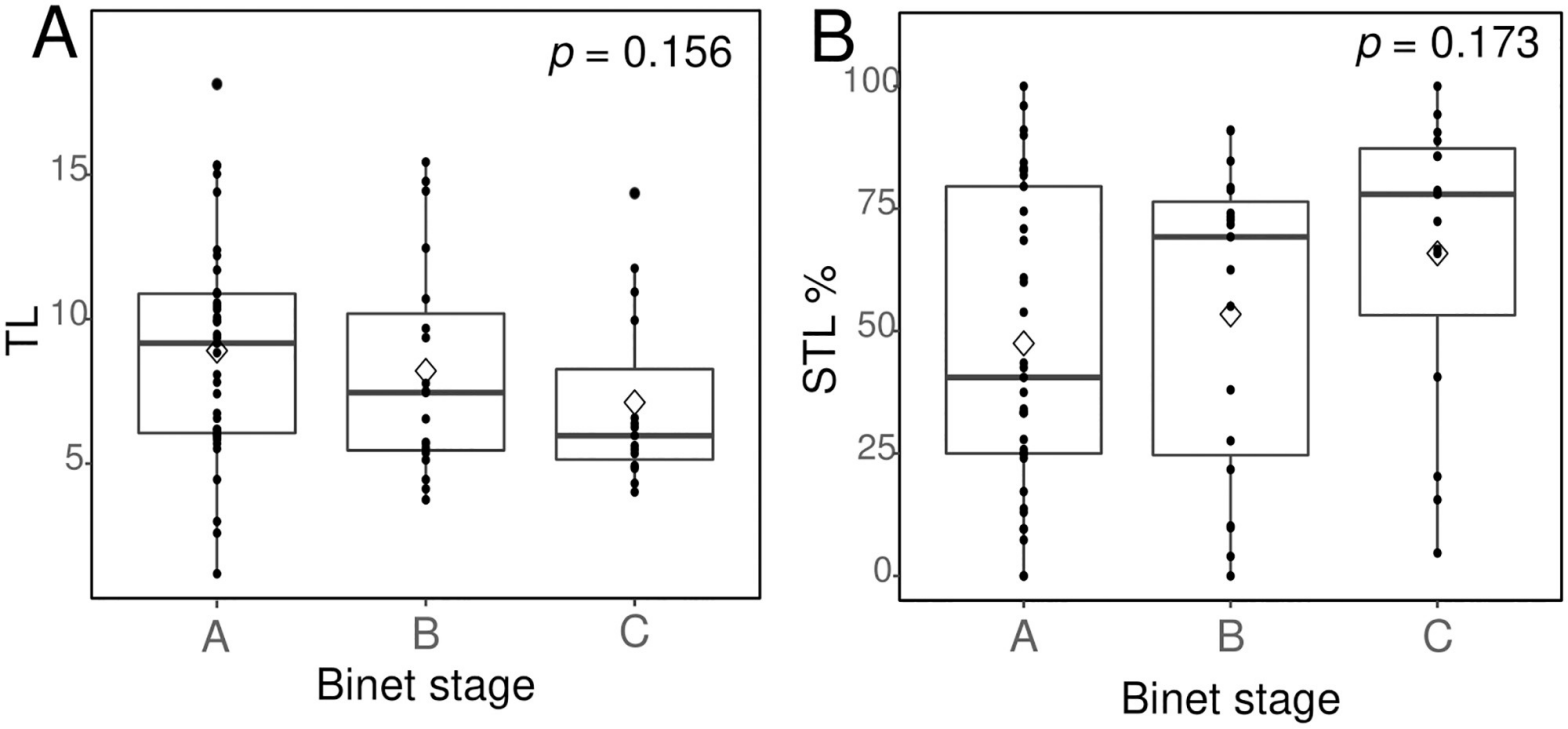
Comparison of TL and the STL% among the Binet stages. A worse Binet stage corresponds to a shorter TL (stage A = 8.91, B = 8.21, C = 7.12, mean values) and a greater STL% (stage A = 47.5, B = 51.4, C = 65.9, mean value). However, no statistically significant differences were found among Binet stages (*p* = 0.156 for TLs, *p* = 0.173 for the STL%).

We performed a correlation analysis between TL and clinical parameters, including age, CBC profile, and BM lymphocyte count (%) (Fig 3). A low Hb level correlated with a short TL (Tb = 0.188, *p* = 0.021) and the STL% (Tb = -0.172, *p* = 0.039). An advanced Binet stage was correlated with shorter TLs (stage A = 8.91, B = 8.21, C = 7.12, mean values) and a higher STL% (stage A = 47.5%, B = 51.4%, C = 65.9%) (Fig 4). However, statistically significant differences among Binet stages (*p* = 0.156 for TLs, *p* = 0.162 for the STL%) were not observed. Among CLL patients who progressed to Richter syndrome, TL was available in only 1 patient, and the mean TL was 4.02, which is markedly shorter than that of Binet stage C (7.12).

**Fig 5:**
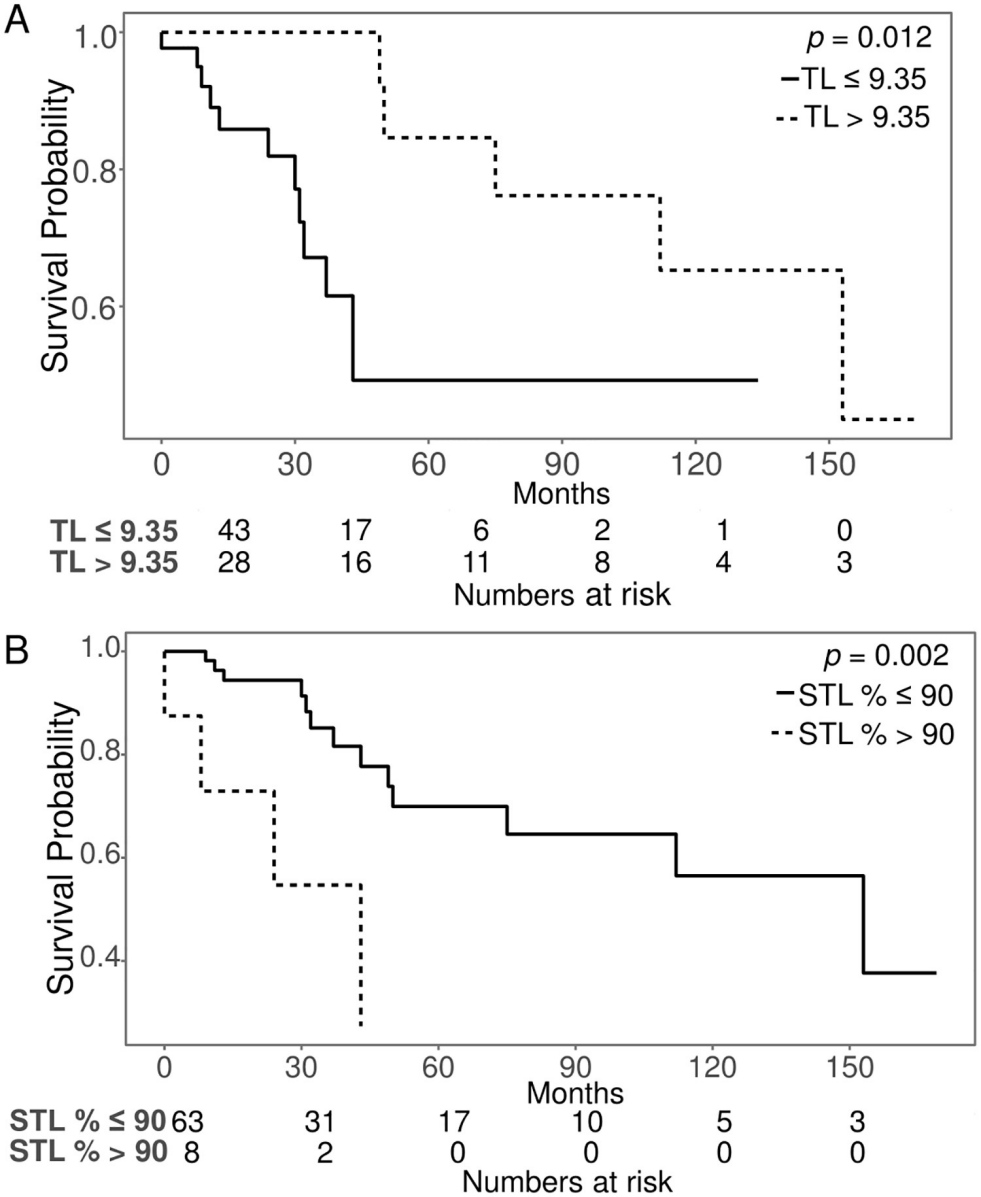
Kaplan-Meier survival curves of TL and the STL%. The overall survival rates changed at a TL of 9.35 and an STL% of 90%.

Regarding TL and survival, patients with a TL of 9.35 or shorter showed significantly lower OS than those with a TL longer than 9.35 (*p* = 0.012). Patients with an STL% greater than 90% showed lower OS than those with an STL% of 90% or less (*p* = 0.002) (Fig 5). In the TTT analysis, patients with a TL of 5.35 or shorter showed significantly lower TTT than did those with a TL longer than 5.35 (*p* = 0.016). Patients with an STL% greater than 54% had a lower TTT than did those with an STL% of 54% or less (*p* = 0.043).

### TL, cytogenetic changes, and somatic mutations

We compared TL according to cytogenetic abnormalities. The genetic mutation analysis of a previous study is described in the S1 Text. The chromosomal aberrations identified in CLL patients were complex karyotypes, trisomy12, del(13q14.3), del(17p13), and del(11q22). Gene mutations detected by multigene target sequencing included *ATM*, *TP53*, *SF3B1*, *KLHL6*, *LAMB4*, *BCOR*, *NOTCH1*, *EZH2*, *CSF1R*, *MYD88*, *SH2B3*, *BRD2*, *FAT4*, *TGM7*, *POT1*, *SF1*, *SETBP1*, *ZRSR2*, *CHD2*, *EGR2*, *MED12*, *RB1*, *LRP1B*, *ITPKB*, *ZMYM3*, *CDKN2A*, *DDX3X*, *STAG2*, *TCF12*, *CEBPA*, *SAMHD1*, *GATA2*, *KIT*, *SCRIB*, *RUNX1*, and *PRKD3* (Table 2). The short TL was associated with a complex karyotype (n = 13) (*p* = 0.030), del(11q22) (n = 8) (*p* = 0.023), and *ATM* and/or *TP53* defect (n = 21) (*p* = 0.019). Patients with complex karyotypes showed lower survival and TTT than those without complex karyotypes (*p* < 0.001, and *p* = 0.014, respectively). Patients with del(17p13) exhibited a lower TTT than did those without del(17p13) (*p* = 0.005). Among genetic variations, *SH2B3* mutation correlated with a short TL (n = 2, *p* = 0.015), but the number of patients with a *SH2B3* mutation was too small to be significant. Patients with *ATM* and/or *TP53* defect also showed lower survival than those without such defects (*p* = 0.003). TL, the STL, del(11q22)/del(17p13), *ATM/TP53* mutation and clinical characteristics (age, stage, progression to Richter syndrome, CBC profile at prognosis) were analyzed by univariate and multivariate Cox analyses for OS and TTT (Table 3, S1 Table). Statistically independent factors of OS were complex karyotypes, *TP53* mutation, and the total number of mutated genes by multivariable Cox analysis. And those of TTT were age, Binet stages B and C, telomere length and the total number of mutated genes.

**Table 2:** Comparison of telomere lengths according to cytogenetic abnormality or gene mutation.

Cytogenetic changes or gene mutation or ZAP70		Number^a^	Mean rank	Sum of ranks	MWU	Z	*p* value
Chromosomal aberration	(-)	48	37.06	1779.0	501.0	-0.627	0.531
	(+)	23	33.78	777.0			
Complex karyotype	(-)	58	38.52	2234.0	231.0	-2.171	**0.030**
	(+)	13	24.77	322.0			
Trisomy 12	(-)	53	36.08	1912.0	420.0	-0.418	0.676
	(+)	17	33.71	573.0			
del(13q14.3)	(-)	40	37.26	1490.5	529.5	-0.837	0.403
	(+)	30	33.15	994.5			
del(17p13)	(-)	66	36.26	2393.0	82.0	-1.265	0.220
	(+)	4	23.00	92.0			
del(11q22)	(-)	63	37.34	2352.5	104.5	-2.271	**0.023**
	(+)	7	18.93	132.5			
*ATM* mutation	(-)	26	18.42	479.0	54.0	-1.629	0.109
	(+)	7	11.71	82.0			
*TP53* mutation	(-)	28	18.04	505.0	41.0	-1.456	0.157
	(+)	5	11.20	56.0			
*SF3B1* mutation	(-)	30	16.70	501.0	36.0	-0.564	0.614
	(+)	3	20.00	60.0			
*KLHL6* mutation	(-)	29	16.48	478.0	43.0	-0.827	0.439
	(+)	4	20.75	83.0			
*LAMB4* mutation	(-)	31	17.39	539.0	19.0	-0.905	0.417
	(+)	2	11.00	22.0			
*BCOR* mutation	(-)	31	16.48	511.0	15.0	-1.207	0.273
	(+)	2	25.00	50.0			
*EZH2* mutation	(-)	31	17.65	547.0	11.0	-1.500	0.200
	(+)	2	7.00	14.0			
*SH2B3* mutation	(-)	31	17.94	556.0	2.0	-2.188	**0.015**
	(+)	2	2.50	5.0			
*FAT4* mutation	(-)	31	16.42	509.0	13.0	-1.358	0.212
	(+)	2	26.00	52.0			
*ATM* defect^b^	(-)	25	19.12	478.0	72.0	-1.581	0.120
	(+)	9	13.00	117.0			
*TP53* defect^c^	(-)	26	18.65	485.0	48.0	-1.894	0.060
	(+)	7	10.86	76.0			
*ATM* and/or *TP53* defect	(-)	20	21.50	430.00	80.0	-2.333	**0.019**
	(+)	15	13.33	200.00			
ZAP70	(-)	20	20.25	405.0	85.0	-1.925	0.056
	(+)	14	13.57	190.0			

Abbreviation: MWU, Mann-Whitney U coefficient

^a^The number of patients according to whether they have certain abnormal karyotypes or mutations

^b^*ATM* defect: del(11q22) and/or *ATM* mutation

^*c*^*TP53* defect: del(17p13) and/or *TP53* mutation

**Table 3:** Univariable and multivariable cox analyses of overall survival among CLL patients^a^.

		Univariable				Multivariable		
Risk factors	Beta	HR	95% CI	*p*	Beta	HR	95% CI	*p*
Age	0.03	1.03	0.99–1.07	0.113				
Stage B	0.24	1.27	0.41–3.92	0.681				
Stage C	0.11	2.92	1.20–7.10	0.018				
Progression to Richter syndrome or not	2.02	7.57	2.77–20.65	< 0.001				
Hemoglobin	-0.11	0.90	0.75–1.08	0.258				
Platelet count > 170,500/μL	-0.64	0.53	0.23–1.17	0.117				
Bone marrow lymphocyte count	0.02	1.02	1.00–1.05	0.033				
Complex karyotype	1.60	4.95	1.78–13.77	0.002	2.46	11.68	2.36–57.90	0.011
Telomere length < 9.35 (T/C ratio)	-0.10	0.90	0.80–1.03	0.118				
STL > 60.9%^b^	0.99	2.68	0.97–7.41	0.058				
Del(11q22) vs. normal	0.65	1.92	0.43–8.54	0.392				
Del(17p13) vs. normal	1.13	3.10	0.89–10.74	0.075				
*ATM* mutation vs. normal	0.76	2.15	0.69–6.72	0.190				
*TP53* mutation vs. normal	1.20	3.31	1.17–9.34	0.024	1.49	4.43	1.40–14.00	0.002
Total mutated gene number	0.40	1.49	1.11–2.00	0.007	0.61	1.84	1.25–2.70	0.002

^a^Factors with *p* value less than 0.2 in the univariate analysis were entered into the multivariate analysis.

^b^STL%: The percentage of cells with the shortest telomere length. The shortest telomere length was defined as less than 7.61 (T/C ratio), which is the 10^th^ percentile TL value of the normal control group.

### TL and *ATM* and *TP53* status

Thirty-three patients were evaluated for del(11q22), *ATM* mutation, and TL. Eight patients harbored *ATM* defects, 5 patients had a biallelic defect of ATM (del(11q22) and *ATM* mutation), 1 patient had del(11q22), and 2 patients had only *ATM* mutations. Patients with a biallelic *ATM* defect had a shorter mean TL than the rest of the patients (T/C ratio: 5.25 in the biallelic *ATM* defect group, 9.23 in the rest of the group, *p* = 0.027). However, survival differences were not observed between patients with biallelic *ATM* defects and the rest of patients by K-M plot of OS and TTT (*p* = 0.287 and *p* = 0.141, respectively). Thirty-two patients were evaluated for del(17p13), *TP53* mutation, and TL. Six patients had *TP53* defects, 2 patients had biallelic defects, 1 patient had only del(17p13), and 3 patients had only *TP53* mutations. Patients with *TP53* defects showed shorter TLs than the *TP53* normal group (T/C ratio: 5.39 in *TP53* defect patients, 9.54 in *TP53* normal patients, *p* = 0.036). K-M plots of OS revealed a survival difference was observed between patients with biallelic *TP53* defects and the rest of patients (*p* = 0.022), while patient with biallelic *TP53* defects displayed no difference in TTT compared to those without biallelic defect (*p* = 0.061). Furthermore, only two patients had a biallelic *TP53* defect, and careful interpretation is necessary. No survival difference was found between the normal *TP53* group and the *TP53* defect group according to the K-M plot of OS and TTT (*p* = 0.075 and *p* = 0.372, respectively).

### Risk factor equation based on Cox analysis

**Fig 6:**
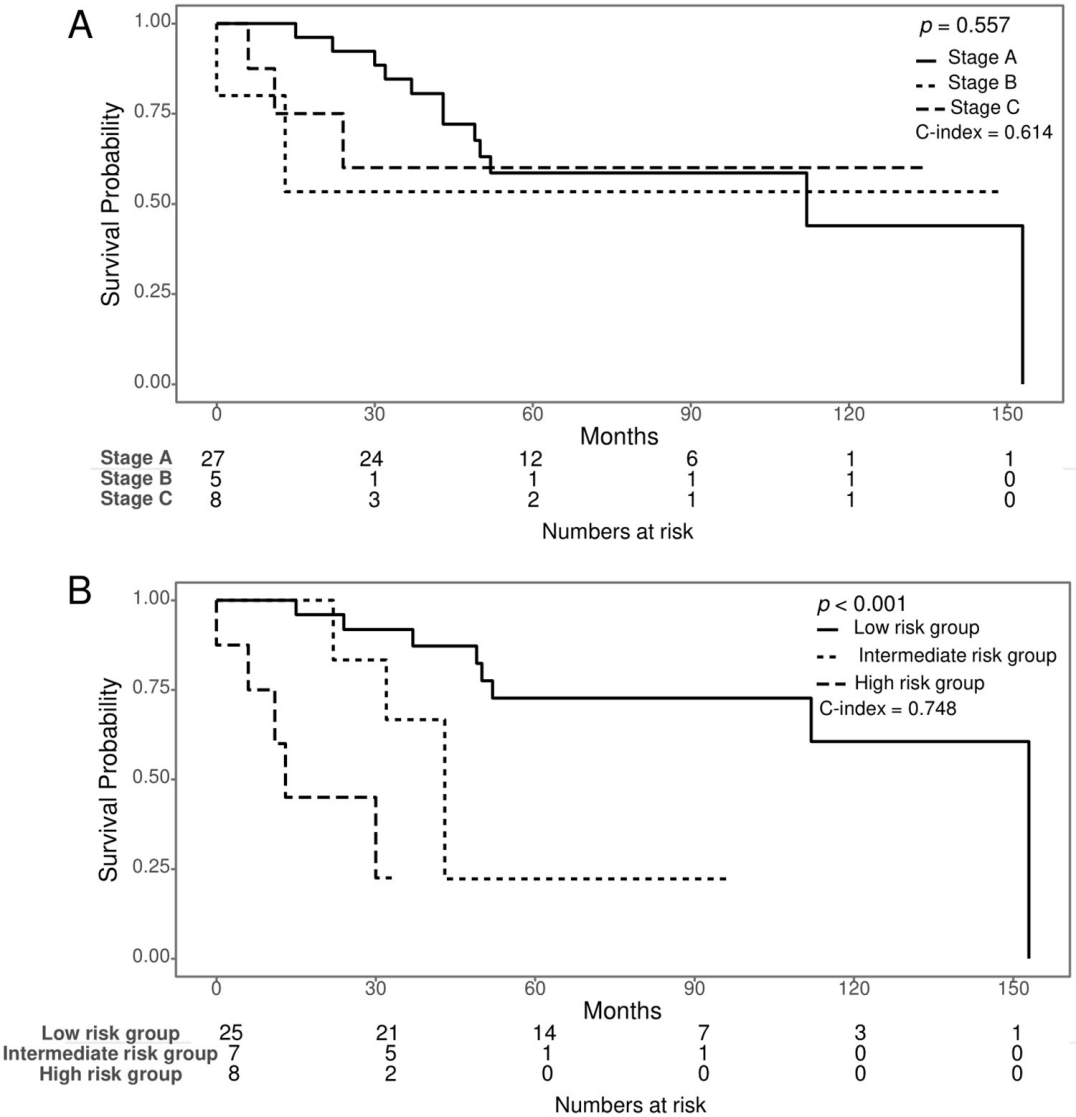
Survival Analysis of the risk equation model compared to Binet stage. (A) In the Kaplan-Meier (K-M) curve, the Binet stage did not show survival differences, and the survival graphs overlapped each other. (B) The risk groups stratified by the risk factor equation of Korean CLL patients showed better K-M survival curves than those stratified by the Binet stage. The risk equation model was complex karyotype × 2.46 + *TP53* mutation × 1.49 + total mutated gene number × 0.61. The low-, intermediate-, and high-risk groups were divided by a risk equation score of 1.83 or less, 1.83 to 3.08, and greater than 3.08, respectively. Harrell’s C-index of risk groups assessed by the risk equation (0.748) was also higher than that of groups stratified by Binet stage (0.624).

For significant variables found in the multivariate analysis of OS, we assigned weighted coefficients for each variable and calculated a risk equation for the Korean model. The risk equation model was complex karyotype (1, 0) × 2.46 + *TP53* mutation (1, 0) × 1.49 + total number of mutated genes × 0.61. When a complex karyotype existed, we applied 1. We divided CLL patients into low-, intermediate-, and high-risk groups based on the risk equation score: 1.83 or less, 1.83 to 3.08, and greater than 3.08, respectively. Binet stage C was the significant risk factor in the univariate analysis (*p* = 0.018); however, the K-M survival graphs overlapped each other (Fig 6A). Stratification of the risk groups by the risk equation model of Korean CLL patients showed clearer discrimination according to the K-M survival curves than that achieved by the Binet stage (*p* < 0.001). Harrell’s C-index of the risk groups assessed by our risk factor equation (0.748 [95% C.I. 0.624–0.872]) was higher than that obtained by assessment according to the Binet stage (0.614 [95% C.I. 0.471–0.758]) (Fig 6B), suggesting that our risk factor equation has superior prognostic ability. With regards to TTT, the risk equation model of TTT was age × -0.12 + TL × -0.26 + total number of mutated genes × 0.73 + stage B (0,1) × 3.3 + stage C (0,1) × 1.99. When the Binet stage was B or C, we applied 1. Harrell’s C-index of the risk groups assessed by our risk factor equation (0.877 [95% C.I. 0.788–0.967]).

## Discussion

In the present study, we investigated the relevance of TL to genomic changes and OS in a Korean population with CLL. As reported in Caucasians, the average TL was a potential predictor of survival in Korean CLL. Distinct from other studies measuring the average TL, we measured TL at the individual cellular level and compared the distribution profiles of TL between the CLL group and a normal control group. The TLs of CLL cells are concentrated in the extremely short TL region, which belongs to the lower 10^th^ percentile region of the TL of the normal control group. Patients with an STL% greater than 90% showed poor survival, suggesting that a higher proportion of cells with extremely attrited telomeres correlates with adverse OS. Meanwhile, with regard to the average TL, we suggest a TL below 9.35 as an adverse biomarker in CLL. Collectively, we presented the values of the average TL (< 9.35 T/C ratio) and STL% (> 90%) as adverse predictors based on Q-FISH. These findings are in line with those of previous studies showing a positive relationship between a short telomere length and risk factors in CLL patients [17,18]. A complex karyotype was an independent adverse prognostic factor by Cox multivariate analysis. Cells with extremely attrited telomeres can undergo chromosomal aberrations such as translocation, deletion, and aneuploidy [19]. In CLL, complex karyotypes are well-known poor prognostic factors in CLL [20]. The present study showed a correlation between complex karyotypes and attrited telomeres, indicating that the occurrence of complex karyotypes results from shortened telomeres. Recent studies have reported that a complex karyotype was associated with advanced disease and poor prognostic markers even in the absence of *TP53*/*ATM* FISH deletion [21]. In the present study, we found a strong association between a short TL and complex karyotypes; a short TL and complex karyotypes were also associated with adverse survival. Among the cytogenetic results in CLL, *TP53* loss is a well-known high-risk factor in addition to *ATM* loss. Our risk stratification models showed that complex karyotypes, *TP53*, and the total number of mutated genes were strong independent predictors.

Thomay et al. measured TL in CLL patients using Q-FISH in a similar manner to our study [22]. To measure TL, they utilized metaphase Q-FISH rather than interphase Q-FISH. Interphase FISH measures TL in a substantial number of cells (200 nucleated cells), while metaphase FISH measures TL in 20 to 30 metaphase cells. Therefore, interphase FISH is considered more representative. However, the results of metaphase FISH can be converted to kb values using software, while interphase FISH results cannot be converted into absolute lengths (kb). Instead, interphase FISH yields TL results expressed as the T/C ratio, which reflects the relative intensity of the telomere signal to the signal intensity of centromere 2. Thomay’s study demonstrated that metaphase TL was shorter in patients with CLL and a complex karyotype than that in healthy controls. Similar to Thomay’s study, the present study also demonstrated a correlation between complex karyotypes and a shorter TL in CLL. Therefore, a correlation between complex karyotypes and a shorter TL in CLL has been identified in both interphase FISH and metaphase FISH.

Recent progress in therapeutics and the discovery of novel prognostic markers in CLL has led to the introduction of a new prognostic scoring system, the CLL-IPI. The CLL-IPI is an index that was designed to integrate existing clinical staging systems with biological and genetic data to refine and provide more specific prognostic information [23]. The CLL-IPI adopts 5 variables: *TP53* status (deleted or mutated), IGHV status, β_2_-microglobulin, clinical stage Binet B/C or Rai I-IV, and age > 65 years. The CLL-IPI study included cytogenetic abnormalities according to FISH (del(17p), del(11q), trisomy 12, del(13q), and del(6q)), but a complex karyotype or any other abnormal karyotype was not considered. A complex karyotype could be recognized as a poor prognostic factor in Korean CLL. The present study did not evaluate the prognostic impact of del(6q), which was evaluated in the CLL-IPI study. However, del(6q) was not included in the CLL-IPI scoring system. del(6q) was reported to be an intermediate prognostic marker in the Chinese population [24].

We also observed an association between a short TL and del(11q.22) in CLL patients. *ATM* and *TP53* deficiency were reported to be associated with a short TL and chromosomal instability [20,25,26]. Since *ATM* and *ATR* signaling regulate the recruitment of human telomerase to telomeres [27], attrition of telomeres in cells with *ATM* defects can result in consequent chromosomal instability. The ATM protein is a central orchestrator of the signal transduction pathway in response to DNA double-strand breaks (DSBs) and regulates checkpoints of the cell cycle and synchronizes DNA repair with the induction of p53-dependent apoptosis [28]. Similar to our study, Rampazzo et al. reported that CLL patients with the 11q, 17p deletion had significantly higher levels of telomerase and shorter telomeres than those with no chromosomal aberration or only 13q deletion [29]. Strefford et al. reported that *TP53* abnormalities and biallelic *ATM* inactivation were found more frequently in the short TL group than in the long TL group [5]. These studies reported an apparent relationship between telomeres and cytogenetic aberrations. When patients have deletion of the *ATM* gene and *ATM* mutation at the same time, we can assume loss of heterozygosity. In the present study, patients with biallelic *ATM* defects and any kind of *TP53* defect, regardless of allelic status, had shorter TLs. Some reports indicate that biallelic defects of *ATM* or *TP53* are associated with poor OS, but the present study did not show such associations [5,30].

Regarding specific genes associated with a short TL, we found an association between a short TL and *SH2B3* mutations. Recently identified molecular markers in CLL include *NOTCH1*, *SF3B1* and *BIRC3* mutations [31,32]. However, *SH2B3* has not yet been reported as a prognostic marker. *SH2B3* (also known as *LNK*) encodes a member of the SH2B3 adaptor family of proteins, which is a key negative regulator of cytokine signaling, plays a critical role in hematopoiesis and lymphoid progenitor, and is expressed at a high level in BM and WBCs [33]]. During B cell lymphopoiesis, SH2B3 linked to the c-Kit receptor acts to inhibit the expansion of an immature population of B cells [34]. We infer that mutated *SH2B3* can eliminate this inhibitory effect, resulting in expansion of an immature B cell population. However, the connection pathway between telomeres and *SH2B3* remains unknown. In solid tumors, genome-wide association studies have identified common susceptibility polymorphisms in colorectal and endometrial cancer near *SH2B3*.

The limitations of our study are as follows. First, because TL was measured by the Q-FISH method and expressed as the T/C ratio, which reflects the relative TL, the TL could not be compared with TLs in previous studies measured in bp using other methods such as terminal restriction fragments or polymerase chain reaction (PCR). The major advantage of interphase Q-FISH is that it can measure the distribution of TL as well as the median or mean TL, while other methods such as southern blot or quantitative PCR can measure only the mean TL. However, quantitative interphase FISH relies on the T/C ratio, which measures the signal of a telomere compared to the centromere signal of chromosome 2 in the same cell. Therefore, interphase FISH cannot measure absolute TL in kb. Second, we did not perform a multiple comparison test because the present study was exploratory research, enrolling small number of patients. Therefore, results with *p* values close to 0.05 should be carefully interpreted.

In conclusion, the TL of Korean CLL patients was related to Hb, del(11q22), *TP53* defects, *ATM* and/or *TP53* defects, and *SH2B3* mutation. Patients with an *ATM* biallelic defect and any *TP53* defect showed shorter TLs than those with monoallelic mutation or deletion. The average TL was a powerful indicator of survival. The STL% may be a supportive prognostic marker. A complex karyotype, which is related to a short TL, was the poorest prognostic factor. TL analysis in CLL is suggested to predict patient outcomes.

## Supporting information

S1 Text Earlier published data on genetic changes in chronic lymphocytic leukemia. (DOCX)

S1 Table Univariable and multivariable cox analyses of Time-to-First-Treatment among chronic lymphocytic leukemia patients. (DOCX)

S1 Fig Distributions of telomere length in chronic lymphocytic leukemia patients and normal controls according to age. (DOCX)
