# Supplementary material for: Telomere length and its correlation with gene mutations in chronic lymphocytic leukemia in a Korean population
Source: PLoS One. 2019 Jul 23;14(7):e0220177. doi: 10.1371/journal.pone.0220177 (PMC6650075; doi:10.1371/journal.pone.0220177)
Supplement: S1 Text — (DOCX) [file pone.0220177.s001.docx]

**Earlier published data on genetic changes in CLL**

Gene mutations were investigated in 48 patients using next-generation sequencing. Three quarters (36/48) of the patients had mutations. Among the patients with mutated genes, the number of detected mutations varied from 1 to 6. More specifically, 29.2% (14/48) of patients had 1 mutation, 22.9% (11/48) of patients had 2 mutations, 14.6% (7/48) of patients had 3 mutations, 4.2% (2/48) of patients had 4 mutations, and 2.1% (1/48) of patients had 5 or 6 mutations. The *ATM* mutation was the most frequent type of mutation (20.8%, 10/48), followed by mutation in *TP53* (14.6%, 7/41), *SF3B1* (10.4%, 5/48), *KLHL6* (8.3%, 4/48), *LAMB4* (6.3%, 3/48), *BCOR* (6.3%, 3/48), and *NOTCH1* (6.3%, 3/48). Mutations in *EZH2*, *CSF1R, MYD88, SH2B3, BRD2, FAT4, TGM7, POT1, SF1, SETBP1, ZRSR2, CHD2, EGR2, MED12, RB1, LRP1B, ITPKB, ZMYM3, CDKN2A, DDX3X, STAG2, TCF12, CEBPA, SAMHD1, GATA2, KIT, SCRIB, RUNX1*, and *PRKD3* were found in less than 5% (1 or 2) of patients. Two or three mutations within one gene were simultaneously observed in the *ATM, TP53, KLHL6, NOTCH1*, and *FAT4* genes of some patients.
