# Supplementary material for: Telomere length and its correlation with gene mutations in chronic lymphocytic leukemia in a Korean population
Source: PLoS One. 2019 Jul 23;14(7):e0220177. doi: 10.1371/journal.pone.0220177 (PMC6650075; doi:10.1371/journal.pone.0220177)
Supplement: S1 Table — (DOCX) [file pone.0220177.s002.docx]

| **S1 Table. Univariable and Multivariable Cox Analyses of Time-to-First-Treatment among Chronic Lymphocytic Leukemia Patients^a^.** | | | | | | | |  |
| --- | --- | --- | --- | --- | --- | --- | --- | --- |
|  |  | Univariable | |  |  | Multivariable | |  |
| Risk factors | Beta | HR | 95% CI | *p* | Beta | HR | 95% CI | *p* |
| Age | -0.02 | 0.98 | 0.96-1.00 | 0.104 | -0.12 | 0.89 | 0.83-0.95 | < 0.001 |
| Stage B | 1.21 | 3.35 | 1.85-6.07 | < 0.001 | 3.30 | 27.03 | 4.21-173.61 | < 0.001 |
| Stage C | 1.65 | 5.18 | 2.79-9.63 | < 0.001 | 1.99 | 7.28 | 1.92-27.69 | 0.004 |
| Progression to Richter syndrome or not | 0.80 | 2.23 | 0.88-5.60 | 0.089 |  |  |  |  |
| Hemoglobin | -0.15 | 0.86 | 0.77-0.96 | 0.006 |  |  |  |  |
| Platelet count > 170,500/µL | -0.49 | 0.62 | 0.38-0.99 | 0.045 |  |  |  |  |
| Bone marrow lymphocyte count | < 0.01 | 1.00 | 0.99-1.01 | 0.740 |  |  |  |  |
| Complex karyotype | 1.00 | 2.72 | 1.41-5.25 | 0.003 |  |  |  |  |
| Telomere length (T/C ratio) | -0.07 | 0.94 | 0.86-1.02 | 0.136 | -0.26 | 0.77 | 0.65-0.91 | 0.003 |
| STL > 60.9%^b^ | 0.52 | 1.68 | 0.89-3.17 | 0.107 |  |  |  |  |
| Del(11q22) vs. normal | -0.06 | 0.94 | 0.33-2.64 | 0.909 |  |  |  |  |
| Del(17p13) vs. normal | 1.24 | 3.45 | 1.44-8.28 | 0.006 |  |  |  |  |
| *ATM* mutation vs. normal | 1.13 | 3.08 | 1.38-6.89 | 0.006 |  |  |  |  |
| *TP53* mutation vs. normal | 0.30 | 1.35 | 0.52-3.50 | 0.541 |  |  |  |  |
| Total mutated gene number | 0.40 | 1.49 | 1.11-2.00 | 0.007 | 0.73 | 2.07 | 1.29-3.31 | 0.003 |
| ^a^Factors with *p* value less than 0.2 in the univariate analysis were entered into the multivariate analysis.  ^b^STL%: The percentage of cells with the shortest telomere length. The shortest telomere length was defined as less than 7.61 (T/C ratio), which is the 10^th^ percentile TL value of the normal control group. | | | | | | | | |
