# Supplementary material for: Telomere length and its correlation with gene mutations in chronic lymphocytic leukemia in a Korean population
Source: PLoS One. 2019 Jul 23;14(7):e0220177. doi: 10.1371/journal.pone.0220177 (PMC6650075; doi:10.1371/journal.pone.0220177)
Supplement: S1 Fig — (DOCX) [file pone.0220177.s003.docx]

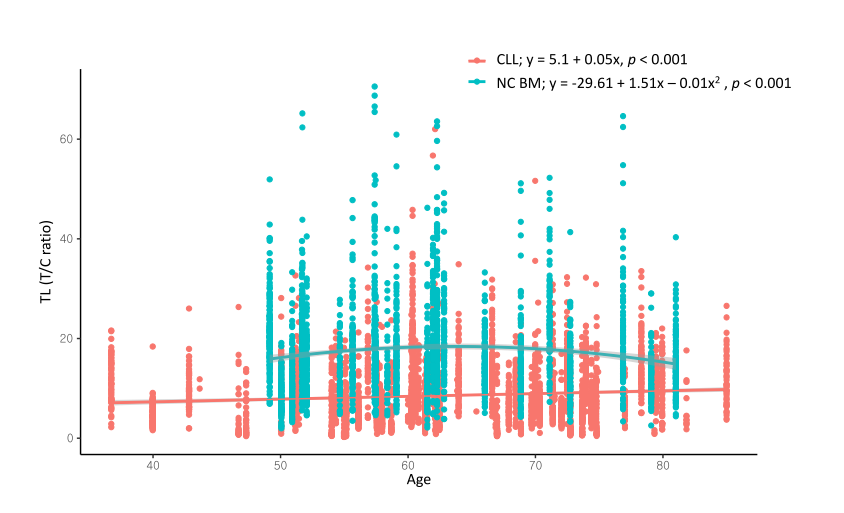


**S1 Fig. Distribution of Telomere Length of Chronic Lymphocytic Leukemia Patients and Normal Controls According to Age.** The telomere length of chronic lymphocytic leukemia patients were shorter than normal controls among all age groups.

Abbreviations: CLL, chronic lymphocytic leukemia; NC, normal control; BM, bone marrow; TL, telomere length.
